# Predictors of injection laryngoplasty volume for glottic insufficiency

**DOI:** 10.1007/s00405-024-08908-2

**Published:** 2024-09-06

**Authors:** Ariel Roitman, Anumitha Venkatraman, Susan Thibeault

**Affiliations:** 1 Division of Otolaryngology-Head and Neck Surgery, Department of Surgery, University of Wisconsin-Madison School of Medicine and Public Health, 600 Highland Avenue, Madison, WI 53792-7375 USA; 2 The Bruce Rappaport Faculty of Medicine, Israel Institute of Technology, Haifa, Israel

**Keywords:** Injection volume, Vocal fold injection, Injection laryngoplasty, Glottic insufficiency

## Abstract

**Purpose:**

Volume injected for glottic insufficiency is paramount in achieving desired outcome. Factors that determine the required volume have not been thoroughly investigated and may correlate with outcome. The first objective of this investigation was to evaluate the association between injectable volume and various parameters, including lifestyle characteristics, pre-procedural factors, and voice measures, while the second aim assessed the correlation of volume to clinical outcomes in patients who underwent injection laryngoplasty.

**Methods:**

For the first objective, a one-way ANOVA and univariate linear regression were used to analyze data from 124 patients (injected material, pre-operative diagnosis, previous voice therapy, age etc.). One-sample t-tests and Pearson correlational coefficients were employed for statistical analysis of aim 2 in a subgroup of 28 patients that had pre- and post-injection voice evaluations (e.g., acoustic and aerodynamic analysis, perceptual assessment, questionnaires).

**Results:**

Average injection volume was 0.39 *±* 0.062 mL (range: 0.1–1.6mL). No pre-procedural or lifestyle factor significantly affected injection volume (*p* > 0.05). There was no relationship between pre-procedural voice outcomes and injection volume (*p* > 0.05). Of the factors that were significantly improved post-injection laryngoplasty (GFI, VHI, and GRBAS), there were no significant correlations between the magnitude of improvement in these measures and injection volume (*p* > 0.05).

**Conclusion:**

Injection volume does not appear to be affected by pre-procedural or lifestyle factors. In addition, injection volume does not significantly impact clinical outcomes assessed through voice analysis or patient-reported questionnaires. Our results underscore the complexity of factors at play in injection laryngoplasty for glottic insufficiency.

## Introduction

Glottic insufficiency, characterized by incomplete closure of the vocal folds during phonation, poses a considerable challenge in the realm of laryngology, significantly impacting voice quality and overall laryngeal function [1]. Despite advancements in therapeutic interventions, the optimal approach to treating glottic insufficiency remains a subject of ongoing investigation. Traditional treatment for glottic insufficiency encompasses a spectrum of options, ranging from voice therapy to surgical interventions [2, 3]. Within this landscape, injection laryngoplasty has gained prominence for its versatility and efficacy. Among the available modalities, injection laryngoplasty has emerged as a valuable tool in the armamentarium, providing a minimally invasive means to augment vocal fold closure and enhance voice outcomes [4].

There are a few augmentation materials that are more routinely used for vocal fold injection for medialization. Hyaluronic acid, calcium hydroxyapatite and fat are popular choices. Patients with glottic insufficiency treatment, that respond favorably to injection laryngoplasty, have improved subjective and possible acoustic outcomes [5, 6].

A critical challenge in injection laryngoplasty application lies in determining the appropriate injection volume, as the success of the procedure is intricately linked to achieving optimal vocal fold augmentation. Recognizing this challenge emphasizes the necessity for a nuanced understanding of predictors influencing the required injection laryngoplasty volume. Individualized treatment strategies are imperative in addressing the heterogeneity of glottic insufficiency presentations. The quest for predictors of injection laryngoplasty volume stems from the recognition that a one-size-fits-all approach may not suffice in achieving optimal outcomes. Additionally, determining the correct injection volume is particularly valuable for patients undergoing general anesthesia, where dynamic interaction and assessment of glottic sufficiency during the procedure are not feasible. The identification of factors influencing the required volume holds the potential to refine treatment planning, enhance procedural precision, and ultimately improve patient satisfaction.

In reviewing the literature, it becomes evident that while injection laryngoplasty has demonstrated effectiveness, studies specifically addressing predictors of injection volume remain limited. Lower injection material amounts (< 0.5 mL) were linked to higher pretreatment S-scores on GRBAS, and a positive correlation exists between injection volumes and Maximum Phonation Time (MPT), in a retrospective study encompassing 186 vocal fold injections, emphasizing the relevance of material quantity in treating glottic insufficiency [7]. A retrospective study assessing 18 patients treated for unilateral laryngeal paralysis found no significant difference in functional outcomes (MPT, mean flow rate, jitter, Voice Handicap Index [VHI], GRBAS) between those receiving less than 0.75 mL of fat (nine patients) and those receiving more than 0.75 mL (nine patients), indicating that the injected volume did not impact overall results [8].

This study aims to fill the existing void in our understanding of predictors for injection laryngoplasty volume in the context of glottic insufficiency. By elucidating these factors, we aspire to pave the way for a more tailored and patient-centered approach to the management of glottic insufficiency, optimizing both therapeutic efficacy and patient-reported outcome measures (PROMs). Our research aims to address two key questions in the context of injection laryngoplasty for vocal fold disorders causing glottic insufficiency. First, we inquire about the pre-procedural factors influencing injectable volume, hypothesizing that characteristics such as the anesthesia type, prior voice therapy, smoking history, injection side (bilateral or unilateral), and Body Mass Index (BMI), may collectively impact treatment effectiveness due to their potential influence on procedural dynamics, glottic efficiency, and tissue resilience. Second, we investigate how injectable volume correlates with various outcome measures, anticipating that it could influence auditory perceptual assessments (GRBAS), acoustic parameters, aerodynamic outcomes, PROMs obtained through questionnaires, and complication rates.

## Materials and methods

This was retrospective analysis of patient data from the University of Wisconsin Madison Voice and Swallow Clinics Outcome Database (IRB #2015 − 0948).

### Inclusion and exclusion criteria

We included adult patients (> 18 years of age), who underwent injection laryngoplasty for glottic insufficiency in an outpatient clinic/in-patient setting. Patients were excluded if data on their injection laryngoplasty treatment were not included in the database (i.e., missing information on date of injection, injection volume etc.). For those patients who received more than one injection laryngoplasty during their care, only information from their first procedure was used for analysis.

### Procedural endpoint definition

Adequate injection was primarily assessed through visualization of medialization. In local anesthesia procedures, the clinician’s perception of the patient’s voice was also considered in some cases to ensure the adequacy of injection.

### General outcome measures

We obtained data on the following factors that may potentially affect injection volume; demographics (age and biological sex (F, M)), diagnosis (glottic insufficiency, scar, atrophy, paralysis, paresis, post intubation phonatory insufficiency), injection material used (Hyaluronic acid or Calcium hydroxyapatite-based), injectable volume (mL), history of prior voice therapy (yes, no), injection side (bilateral, right, left vocal fold), anesthesia type (local vs. general), smoking history (yes, no) and BMI.

### Voice outcome measures

We obtained data on pre-injection and post-injection voice measures, obtained at office visits that flanked an injection laryngoplasty treatment. Measures included Glottal Function Index (GFI) (4-item assessment measuring a patient’s self-perceived vocal effort, fatigue, quality, and pain), VHI (30-item assessment that delineates the self-perceived to effect of a voice problem on an individual’s quality of life). Acoustic voice measures included MPT, jitter, shimmer, maximum fundamental frequency (Fo high), lowest pitch and intensity, and Dysphonia Severity Index. Aerodynamic measures included Phonation threshold pressure, subglottal pressure, aerodynamic resistance, and glottal airflow. Auditory perceptual measures included GRBAS ratings (G = Grade of overall severity of dysphonia, R = Roughness, B = Breathiness, A = Asthenia, and S = Strain voice quality). For the subset of patients where both pre-injection and post-injection voice measures could be obtained (*n* = 28), voice measure change was calculated by subtracting post-injection – pre-injection values.

### Statistical analysis

In aim one to determine whether the injection volume was affected by the following categorical variables - diagnosis, smoking history, injection material, anesthesia type, prior voice therapy, BMI – we completed individual one-way ANOVAs to compare injection volume across within-factor levels (*n* = 124 patients). Next, we completed univariate linear regression analyses for each pre-injection voice outcome measure (listed in the Voice Outcome Measures section) as well as the continuous variables (age) to determine if these measures predicted the injection volume for each procedure (*n* = 124 patients). If a patient did not have data on a certain factor, they were removed from the analysis of that factor (for example, number of cigarette packs were reported by only 22 patients). For aim 2 we completed one-sample t-tests, for patients wherein voice measures were obtained pre- and post-injection (*n* = 28), to determine if voice measures improved post-procedure. As a follow up, we completed Pearson’s correlation coefficients between injection volume and those voice measures that improved post-injection (*n* = 28).

## Results

### Patient characteristics

124 patients met inclusion criteria for Aim 1 (mean age 62.88, [SD 15.08] at time of injection, 69.6% male [*n* = 71]), average BMI 29.45 [SD 11.8]). Average injection volume across all procedures was 0.39 mL [SD 0.062]. Of these patients, five were placed under general anesthesia prior to the procedure (4%), fourteen had received prior voice therapy (13.7%), seventeen received a right-sided injection only (16.66%), forty-nine received a left-sided injection only (48.14%), and the remaining received a bilateral injection (*n* = 36, 35.2%). The following injection materials were used; calcium hydroxyapatite (CaHa, *n* = 51) and hyaluronic acid (*n* = 73). This was subdivided into (Restylane *n* = 8, Cymetra *n* = 9, Hylaform *n* = 10, Renu *n* = 13, Radiesse *n* = 21, Juvederm *n* = 63). The following otolaryngology diagnoses were included; vocal fold scar (*n* = 8, 6.4%), vocal fold paresis (*n* = 18, 14.5%), vocal fold atrophy (*n* = 27, 21.7%), post-intubation phonatory insufficiency (*n* = 1, 0.9%), vocal fold paralysis (*n* = 70, 56.4%). Of these patients, twenty-eight patients completed pre-injection and post-injection voice evaluations.

### Aim 1 - evaluation of variables impacting injection volume

There were no significant effects for any of the categorical variables on one-way ANOVAs; glottic insufficiency etiology (*p* = 0.239), sex (*p* = 0.079), injection material (*p* = 0.493), anesthesia type (*p* = 0.896), prior voice therapy (*p* = 0.882), smoking history (*p* = 0.783), cigarette packs (*p* = 0.501), injection side (*p* = 0.394), and BMI (*p* = 0.695). There was no significant effect on univariate linear regression for the continuous variable age (*p* = 0.238), or voice measures; MPT (*p* = 0.103), Fo high (*p* = 0.610), lowest pitch (*p* = 0.860), lowest intensity (*p* = 0.223), jitter (*p* = 0.255), Dysphonia Severity Index (*p* = 0.356), Phonation Threshold Pressure - PTP (*p* = 0.434), subglottal pressure (*p* = 0.998), glottal airflow (*p* = 0.240), aerodynamic resistance (*p* = 0.572), GFI (*p* = 0.332), VHI (*p* = 0.113), and GRBAS (G(*p* = 0.189), R(*p* = 0.171), B(*p* = 0.521), A(*p* = 0.164), S(*p* = 0.805), and BMI (*p* = 0.695). Table 1 depicts the described data.

**Table 1:** Analyzed potential predictors of injection laryngoplasty volume for glottic insufficiency (aim 1 assessment)

Assessed parameter	Statistic (t- or F-test, *p*-value)
Glottic insufficiency etiology	F (3,123) = 1.248, *p* = 0.239
Sex (Male vs. Female)	t (118) = -1.424, *p* = 0.079
Age	F (1,26) = 1.459, *p* = 0.238
Smoking history (Yes vs. No)	F (3,26) = 0.360, *p* = 0.783
Cigarette packs (Packs per year)	F (1,21) = 0.428, *p* = 0.501
Body Mass Index (kg/m^2^)	F (1,65) = 0.936, *p* = 0.695
Prior voice therapy (Yes vs. No)	F (1, 101) = 0.022, *p* = 0.882
Anesthesia type (Local vs. General)	F (1,123) = 0.017, *p* = 0.896
Injection material (Hyaluronic acid vs. Calcium hydroxyapatite based)	F (3,123) = 2.243, *p* = 0.493
Injection side (Right vs. Left vs. Bilateral)	F (2,123) = 0.666, *p* = 0.394
Maximum Phonation Time (Seconds)	F (1, 26) = 3.240, *p* = 0.103
Maximum fundamental frequency (Hertz)	F (1, 26) = 0.267, *p* = 0.610
Lowest pitch (Hertz)	F (1, 26) = 0.32, *p* = 0.860
Lowest intensity (dB)	F (1, 26) = 1.558, *p* = 0.223
Jitter (Unit Interval)	F (1, 26) = 1.363, *p* = 0.255
Phonation Threshold Pressure (cmH_2_O)	F (1, 26) = 0.644, *p* = 0.434
Subglottal pressure (cmH_2_O)	F (1, 26) = 0.001, *p* = 0.998
Glottal airflow (m^3^/s)	F (1, 26) = 1.488, *p* = 0.240
Aerodynamic resistance (Newtons)	F (1, 26) = 0.333, *p* = 0.572
Dysphonia Severity Index	F (1, 26) = 0888, *p* = 0.356
Glottal Function Index	F (1, 26) = 0.985, *p* = 0.332
Vocal Handicap Index	F (1, 26) = 2.717, *p* = 0.113
GRBAS	G score (F (1,26) = 1.833, *p* = 0.189)
	R score (F (1,26) = 1.996, *p* = 0.171)
	B score (F (1,26) = 0.425, *p* = 0.521)
	A score (F (1,26) = 2.070, *p* = 0.164)
	S score (F (1,26) = 0.063, *p* = 0.805)

GRBAS = G for Grade, R for Roughness, B for Breathiness, A for Asthenia, and S for Strain

### Aim 2 – assessment of post-procedural voice measures improvement

Independent of volume-related considerations, our findings indicate that among all voice outcome measures, MPT (*p* = 0.002) and highest pitch (*p* = 0.014) both increased post-injection laryngoplasty. In addition, GFI (*p* < 0.001), and VHI (*p* < 0.001) also improved post-injection (p *≤* 0.05). There were significant changes for ratings for G (t(27) = 2.5, *p* = 0.019), and B (t(27) = 3.309, *p* = 0.003) within GRBAS. There were no significant changes for other voice outcomes post-injection; lowest intensity (*p* = 0.582), jitter (*p* = 0.427), lowest pitch (*p* = 0.253), Dysphonia Severity Index (*p* = 0.744), PTP (*p* = 0.119), subglottal pressure (*p* = 0.243), glottal airflow (*p* = 0.712), aerodynamic resistance (*p* = 0.163) and ratings of R (t(27) = 1.619, *p* = 0.107), A (t(27) = 1.968, *p* = 0.06), and S (t(27) = 1.331, *p* = 0.195) within GRBAS (Table 2).

**Table 2:** Overall pre- and post-injection laryngoplasty voice measures analysis

Assessed parameter	Pre-injection (Mean *±* SD)	Post-injection (Mean *±* SD)	Statistic (t-test, *p*-value)
MPT (Seconds)	8.25 *±* 2.42	15.54 *±* 3.01	t (27) = -3.020, *p* = 0.002
Lowest pitch (Hertz)	135.34 *±* 33.97	63.59 *±* 6.87	t (27) = 1.16, *p* = 0.253
Lowest Intensity (dB)	70.23 *±* 17.63	66.35 *±* 15.79	t (27) = 0.555, *p* = 0.582
Highest Pitch (Hertz)	385.24 *±* 159.25	428.48 *±* 178.18	t (27) = -2.284, *p* = 0.014
Jitter (Unit Interval)	6.217 *±* 13.08	2.023 *±* 2.08	t (27) = 0.802, *p* = 0.427
PTP (cmH_2_O)	6.1 *±* 3.5	5.56 *±* 2.72	t (27) = -1.202, *p* = 0.119
Subglottal pressure (cmH_2_O)	8.4 *±* 2.03	8.38 *±* 3.57	t (27) = 1.183, *p* = 0.243
Glottal airflow (m^3^/s)	0.68 *±* 1.77	0.17 *±* 0.99	t (27) = 0.372, *p* = 0.712
Aerodynamic resistance (Newtons)	54.49 *±* 110.04	60.2 *±* 41.14	t (27) = -1.416, *p* = 0.163
DSI score	-1.23 *±* 25.73	-2.67 *±* 2.94	t (27) = 0.330, *p* = 0.744
GFI score	13.76 *±* 3.77	6.85 *±* 5.88	t (27) = 4.042, *p* < 0.001
VHI score	65.56 *±* 25.33	60.19 *±* 41.14	t (27) = 3.962, *p* < 0.001

MPT = Maximum Phonation Time, PTP = Phonation Threshold Pressure, DSI = Dysphonia Severity Index, GFI = Glottal Function Index, VHI = Vocal Handicap Index

Turning to the volume-related analysis, there were no significant correlations between voice measures that improved post-injection and injection volume; MPT (*r* = 0.028, *p* = 0.869), highest pitch (*r* = 0.273, *p* = 0.097), GFI (*r* = 0.332, *p* = 0.113), VHI (*r* = 0.27, *p* = 0.332), and ratings of G (*r* = 0.031, *p* = 0.878), B (*r* = 0.009, *p* = 0.964) within GRBAS.

## Discussion

The present study sought to investigate the relationship between injectable volume and various parameters in injection laryngoplasty for vocal fold disorders causing glottic insufficiency.

In examining pre-procedural factors influencing injectable volume (Aim 1), our results indicated that pre-procedural diagnosis, injectable material, anesthesia type, prior voice therapy, smoking history and quantity, injection side and BMI did not exhibit a discernible impact on the volume of filler used during the procedure. Furthermore, no correlation was observed with auditory perceptual assessments, acoustic and aerodynamic parameters, and PROMs. Indeed, the absence of correlation reinforces the idea that there are various influences at play or inherent variations in patient responses including how tissues interact with the filler, the way the filler disperses within the tissue, and individual healing dynamics.

Several variables mentioned earlier have been examined in the broader context of literature on injection laryngoplasty. There was no significant correlation observed between voice parameters and BMI following fat injection [9]. Regarding anesthesia type, injection laryngoplasty under local anesthesia yields comparable results to procedures performed under general anesthesia in terms of voice outcomes and patient satisfaction [10, 11]. Additionally, Miyata assessed the impact of early voice therapy on patients with unilateral vocal fold paralysis following esophagectomy. Comparing between a voice therapy and non-voice therapy groups, results showed notable improvements in subglottal pressure, mean flow rate, and maximum phonation time in the voice therapy group, indicating that voice therapy may be effective in enhancing vocal function in unilateral vocal fold paralysis patients [12].

Shifting to the impact of injectable volume on outcome measures, a review of the literature elucidates changes in various outcome parameters following injection laryngoplasty. In a prospective study analyzing data from 75 patients who received single hyaluronate injection laryngoplasty the authors concluded that hyaluronate injection laryngoplasty improved glottal conformation, aerodynamics, and voice [13]. Auditory perceptual assessments and PROMs were also assessed in the literature, Birken et al., in a prospective study, discovered that injection laryngoplasty is an effective method of treating glottal insufficiency, as measured by VHI-30; Consensus Auditory-Perceptual Evaluation of Voice (CAPE-V); and vocal Grade, Roughness, Breathiness, Asthenia and Strain (GRBAS) which were evaluated prior to in-office injection and 2 months postinjection [14]. PROMs and perceptual measurements were shown to improve after injection laryngoplasty in more studies [7, 15, 16]. Acoustic and aerodynamic outcomes have been extensively investigated in the literature and found to be equivocal, some observed significant improvement with injection laryngoplasty while other showed partial improvement [17–19].

With that being noted, the vast majority of studies have not rigorously directed their focus toward evaluating injectable volume as a predictor for voice outcomes. One study that addressed volume as a variable assessed vocal function and glottal form improvement after autologous fat injection laryngoplasty in 73 patients with unilateral vocal fold paralysis. Patients were categorized into low-volume (< 3 mL) and high-volume (≥ 3 mL) groups. Results indicated that injecting more than 3 mL of autologous fat into the vocal muscle layer is recommended for reliable enhancement of voice function, with the high-volume group showing more significant improvements in some aerodynamics evaluations, pitch and intensity measurements, and acoustic analyses [20]. Arguably, these findings may not be generalized to off-the-shelf fillers, given the substantial volume difference required for fat compared to other products. Our study delved into a broader spectrum of commercial injected materials commonly used globally to provide a more universally applicable recommendation.

Evaluating the influence of injectable volume on outcome measures (Aim 2), our study demonstrated no significant correlation with auditory perceptual assessments (GRBAS), acoustic parameters, aerodynamic outcomes, and PROMs. Some studies advocate for a standardized injection of 1 mL [21, 22] or 2 mL [23] of hyaluronic acid guided by laryngeal electromyography, asserting that it leads to vocal fold overcorrection. They propose that the shape of the over-injected vocal fold remodels in response to compression from the contralateral mobile vocal fold, resulting in favorable outcomes. Our finding challenges such assertions about the impact of uniform injectable volume on treatment outcomes, suggesting that tailored treatment is likely necessary for this procedure rather than a one-size-fits-all approach. These findings reinforce avenues for discussions on the complex interplay of variables affecting treatment efficacy and highlights the need for a more nuanced understanding of the factors influencing patient response to injection laryngoplasty.

While our study provides satisfactory insights, several limitations must be acknowledged. The sample size may not be large enough to eliminate some biases and the inherent variability in patient characteristics that may have influenced the robustness of the results. Furthermore, the study’s retrospective nature could introduce biases, and selected parameters may not fully capture the complexity of the treatment’s effectiveness. In pursuit of our second aim, the analysis centered on patients with pre- and post-injection voice evaluations, encompassing diagnoses of vocal fold atrophy, vocal fold paralysis, and paresis. Upon delving into detail, cases of glottic insufficiency resulting from vocal fold paralysis may exhibit improvement due to favorable synkinesis, leading to increased variability. Similarly, cases involving vocal fold scars, contributing to hoarseness through both an increased glottic gap and secondary factors like stiffness and abnormal vocal fold vibration, may also introduce variability. The initial goal of reducing biases with a sizable patient cohort proved unattainable, underscoring the potential benefits of an increased sample size for strengthening the study’s validity and mitigating other potential biases. Finally, our study did not include acoustic analysis techniques such as the cepstral spectral index of dysphonia (CSID) and cepstral peak prominence (CPP). Incorporating these methods in future studies could provide additional insights into the relationship between injectable volume and voice outcomes in injection laryngoplasty.

In light of these acknowledged limitations, the study’s findings, indicating a lack of correlation, underscore the value of tailoring treatment to individual needs rather than adhering to a uniform procedure. Clinicians should approach pre-procedural planning and post-procedural expectations with caution, considering the individualized nature of patient responses to injection laryngoplasty. Future research endeavors should focus on refining study methodologies, exploring additional variables, and expanding the scope to enhance our understanding of the intricate factors influencing treatment outcomes in glottic insufficiency.

## Conclusion

Our study marks the first comprehensive investigation into the relationship between injectable volume of a wide range of commercial materials and pre- and post-procedural aspects in injection laryngoplasty for glottic insufficiency. Significantly, our findings indicate that neither pre-procedural elements nor lifestyle factors significantly influence the volume of filler used during injection laryngoplasty. Furthermore, we found no significant correlation between injectable volume and various outcome measures, including auditory perceptual assessments, acoustic and aerodynamic parameters, and patient-reported outcomes. These results underscore the intricate nature of injection laryngoplasty and highlight the importance of tailored approaches in treating glottic insufficiency.
